# Cost savings associated with timely treatment of botulism with botulism antitoxin heptavalent product

**DOI:** 10.1371/journal.pone.0224700

**Published:** 2019-11-07

**Authors:** Deborah M. Anderson, Veena R. Kumar, Diana L. Arper, Eliza Kruger, S. Pinar Bilir, Jason S. Richardson

**Affiliations:** 1 Emergent BioSolutions Canada Incorporated, Winnipeg, Manitoba, Canada; 2 Emergent BioSolutions Incorporated, Gaithersburg, Maryland, United States of America; 3 IQVIA, San Francisco, California, United States of America; Instituto Butantan, BRAZIL

## Abstract

**Background:**

Botulism is a rare, serious, and sometimes fatal paralytic illness caused by exposure to neurotoxins produced by *Clostridium botulinum* bacteria. Patients with documented or suspected exposure to botulinum toxin serotypes A-G can be treated with BAT^®^ [Botulism Antitoxin Heptavalent (A, B, C, D, E, F, G)–(Equine)] product, which was approved in 2013 in the United States (US). Patients with botulism have demonstrated greater clinical benefit with early BAT product treatment (≤2 days from symptom onset) versus late treatment (>2 days).

**Objective:**

Economic outcomes associated with improved clinical outcome benefits of BAT product treatment have not yet been reported. This *ad hoc* analysis aimed to estimate and compare costs associated with hospitalization, intensive care unit stay, and mechanical ventilation for patients with botulism administered BAT product treatment early or late.

**Methods:**

Clinical outcomes data for early and late BAT product treatment were obtained from a patient registry conducted between October 2014 and July 2017. Total per patient mean daily costs were estimated based on information from published literature. Total population costs per group were calculated by multiplying estimated mean cost per patient by the average annual number of non-infant botulism cases in the US.

**Results:**

Mean per patient costs were 2.5 times lower for patients treated with BAT product early versus late. On average in the US, early BAT product treatment could save greater than $3.9 million per year versus late treatment.

**Conclusion:**

Substantial economic savings can be achieved with early BAT product treatment. The findings support the recommendation for public health authorities to ensure antitoxin treatment is readily available in sufficient quantities to manage botulism cases, including sporadic outbreaks and potential mass exposure biological attacks.

## Introduction

Botulism is a rare, serious, and sometimes fatal paralytic illness caused by exposure to neurotoxins produced primarily by *Clostridium botulinum*, an anaerobic Gram-positive bacterium [1]. Between 2001 and 2016 more than 2,400 botulism cases in the United States (US) were reported to the Centers for Disease Control and Prevention (CDC), averaging over 150 per year [2]. There are seven known serotypes of botulinum toxin, designated by the letters A through G; in humans, botulism is primarily caused by serotypes A, B, E, and sometimes F [3]. These neurotoxins have been described as the most lethal substances known to science [4, 5]. The disease is categorized according to exposure and transmission route, i.e., foodborne, wound, infant (often intestinal colonization), adult intestinal toxemia, iatrogenic, and inhalational botulism [6].

The pathophysiology of the disease involves absorption of botulinum toxin into the circulation with uptake into neuromuscular junctions that inhibits release of acetylcholine. While the time course of clinical onset following exposure may vary by route and amount of toxin exposure, the signs and symptoms of botulism remain the same [4]. These include vision problems, difficulty breathing and swallowing, generalized weakness, and paralysis [7]. Involvement of the muscles of respiration can lead to respiratory failure and death, if untreated [8].

Historically, the mortality rate for patients with botulism was as high as 60% [9–11]. Standard therapy for patients with botulism primarily consists of supportive care, including mechanical ventilation (MV) as required, and passive immunization with an antitoxin [12]. With current respiratory and intensive care practices as well as availability of botulism antitoxins the mortality rate has dropped to less than 7% [12]. Although the mortality rate has decreased, the length of stay (LOS) in the hospital or in the intensive care unit (ICU) required for patients to recover, in addition to the need for extended duration of MV, can result in significant medical costs [13, 14].

Currently in the US, there are two available licensed products to treat botulism; BabyBIG^®^ [Botulism Immune Globulin Intravenous (Human) (BIG-IV)], which is used exclusively to treat botulism in infants (i.e., children under one year of age) caused by toxin serotypes A and B [15], and BAT^®^ [Botulism Antitoxin Heptavalent (A, B, C, D, E, F, G)–(Equine)], which was US Food and Drug Administration (FDA)-approved in 2013, and is indicated for the treatment of symptomatic botulism following documented or suspected exposure to botulinum neurotoxin serotypes A, B, C, D, E, F, or G in adults and pediatric patients [16]. BAT product is a sterile solution of purified F(ab')_2_ plus F(ab')_2_-related immune globulin fragments prepared from plasma obtained from horses that have been immunized with a specific serotype of botulinum toxoid and toxin. To obtain the final heptavalent product, the seven antitoxin serotypes are blended. BAT product is supplied in a 50-milliliter vial size. A single adult dose of BAT product is one vial administered intravenously; a pediatric dose of BAT product is 20% to 100% of an adult dose based on body weight of the pediatric patient or 10% of an adult dose regardless of body weight for infants. Each single-use vial contains a minimum antitoxin potency of 4,500 Units (U) serotype A antitoxin; 3,300 U serotype B antitoxin; 3,000 U serotype C antitoxin; 600 U serotype D antitoxin; 5,100 U serotype E antitoxin; 3,000 U serotype F antitoxin; and 600 U serotype G antitoxin [16]. The product potency is expressed in units based on the mouse neutralization assay. Each unit of BAT product is designed to neutralize 10,000 mouse intraperitoneal lethal dose 50% units (MIPLD_50_) of botulinum neurotoxin for serotype A, B, C, D, F, and G and 1,000 MIPLD_50_ of serotype E [16]. BAT product is the only US FDA-approved drug for the treatment of botulism in both adult and pediatric (including infant) patients that targets known toxin serotypes A-G [16]. In the US, BAT product is distributed from the Strategic National Stockpile (SNS) for suspected botulism cases [17].

Clinical experience with BAT product was reported by the CDC under a pre-licensure expanded-access (EAP) Investigational New Drug (IND) program conducted from March 2010 through March 2013 [18]. Among patients with confirmed botulism, those treated early with BAT product (≤2 days from botulism symptom onset) spent significantly fewer days in the hospital and the ICU than those treated late (>2 days from symptom onset) [18]. Similar findings were observed under a patient registry established following US licensure of BAT product. Registry patients with botulism who received treatment with BAT product ≤2 days from botulism symptom onset were hospitalized for a shorter time and spent less time in the ICU, fewer patients required MV and if required, were mechanically ventilated for a shorter duration than patients who received late treatment [19]. In both the CDC’s EAP and the patient registry, improved morbidity and mortality rates were observed with early BAT product treatment and no deaths were BAT product-related [18, 19].

A recent retrospective study looked at medical records and billing information collected for US patients treated with BabyBIG for infant botulism from 2003 to 2015. That study concluded that the use of BabyBIG led to shortened hospital stay and resulted in a mean decrease in hospital charges of $88,900 USD per patient, with more than $85 million in hospital charges avoided overall during that time period. Additionally, cost differences were time-dependent; treatment beyond the seventh hospital day led to longer mean hospital stays and correspondingly higher charges than treatment within the first seven days of hospital admission [20].

While it has been established that BAT product is safe and early treatment results in greater clinical outcomes benefit than delayed treatment, the potential economic costs or savings associated with treatment at different timepoints have not yet been quantified. The objective of this *ad hoc* analysis was to estimate the various costs associated with hospitalization, ICU stay, and MV in conjunction with early (≤2 days of symptom onset) BAT product treatment or late (>2 days from symptom onset) BAT product treatment.

## Methods

### Analysis overview

A comparative analysis of costs associated with clinical outcomes of treating patients with BAT product early or late was performed from the US hospital perspective using a model developed in Microsoft^®^ Excel 2016. The model population included pediatric and adult patients with symptomatic botulism, where early treatment was defined as BAT product administration ≤2 days from time of botulism symptom onset and late treatment was defined as BAT product administration >2 days from time of symptom onset.

For each treatment timing group, the model estimated the total per patient mean costs associated with the acute (i.e., inpatient) phase of care as well as total costs for a hypothetical annual caseload in the US population. At the patient level, clinical outcomes associated with the acute phase of care (LOS in the general ward [non-ICU], ICU LOS, and duration of MV) were multiplied by respective estimated *per diem* costs (USD 2018) to capture the total cost to manage an average patient with symptomatic botulism. Total estimated population costs per treatment timing group were calculated by multiplying mean cost per patient by the average annual number of non-infant confirmed and probable botulism cases in the US. The annual number of cases (n = 69) was based on the average of non-infant confirmed and probable cases reported in 2015 (n = 72) and 2016 (n = 65) (the two most recent years for which US surveillance summary data were available) [2]. Infant cases of botulism were excluded from the calculations based on the assumption that US infants are more likely to be treated with BabyBIG product given its history of having been the only available product for use in infants before licensure of BAT product. Infection in infants is caused by ingestion and subsequent pathogen proliferation because at this young age protective intestinal flora are too underdeveloped to inhibit harmful bacterial colonization. Although BabyBIG only covers toxin serotypes A and B, infant botulism is primarily caused by these two serotypes [21, 22].

### Data sources

Hospitalization parameters (total hospital LOS, ICU LOS, MV duration) for early and late BAT product treatment were obtained from the Emergent BioSolutions Canada Inc. (EBCI)-sponsored non-interventional, retrospective, phase 4 registry designed to evaluate safety and capture clinical outcomes of patients treated with BAT product in the US (ClinicalTrials.gov Identifier: NCT02055183). Data were collected between October 2014 and July 2017, during which time 162 patients were enrolled in the registry; 113 (69.8%) of these patients had a final diagnosis of botulism [19]. Among the patients who did not have botulism, 24 (14.8%) were reported to have a final diagnosis of Guillain-Barré syndrome, 3 (1.9%) patients had myasthenia gravis, 2 (1.2%) patients had stroke/central nervous system mass/lesion, and 2 (1.2%) patients had multiple diagnoses. For the remaining 18 (11.1%) patients, the diagnosis was described as “other” or “unknown” or was not reported [19].

Among the patients with a final diagnosis of botulism, the median age (range) was 47 years (32 days to 92 years) and 68 (60.2%) were male. Fifty-seven (50.4%) patients had foodborne botulism. Wound botulism occurred in 28 (24.8%) patients, while iatrogenic botulism and intestinal colonization (infant or adult) were reported in 2 (1.8%) patients each. For 24 (21.2%) patients, the cause of botulism was unknown or was not included in the clinical report form [19].

Among the 113 patients with a final diagnosis of botulism, 107 had adequate data to determine whether they received early versus late treatment. The patients who received early BAT product treatment (n = 45) were older (median age 56 years; range two to 88 years) than those who received late treatment (n = 62); (median age 41 years; range 32 days to 92 years). Patients who were treated early comprised fewer males (51.1%) than those treated late (67.7%). There was a higher incidence of foodborne botulism among patients treated early (68.9%) than those treated late (40.3%) as well as a slightly higher incidence of iatrogenic botulism (2.2% early; 1.6% late) among patients treated early, while incidences for wound botulism (15.6% early; 27.4% late), infant or adult intestinal colonization (0 early; 3.2% late), and unknown or not reported (13.3% early; 27.4% late) were lower among patients treated early [19].

Clinical outcome values (mean and 95% confidence intervals) for patients with a final diagnosis of botulism and known early vs late treatment are reported in Table 1. Total hospital LOS was composed of non-ICU LOS and ICU LOS.

**Table 1:** Mean values for clinical outcome parameters.

Clinical Outcome Parameter	Early BAT Product Treatment (Registry; n = 45)		Late BAT Product Treatment (Registry; n = 62)	
	Mean Days	95% CI	Mean Days	95% CI
**Total Hospital LOS**	11.4	7.4–15.5	24.2	18.0–30.4
** Non-ICU LOS**^**a**^	4.3	2.8–5.7	8.6	7.7–9.6
** ICU LOS**	7.2	4.6–9.7	15.6	10.2–20.9
**MV Duration**	3.7	2.1–5.3	17.3	9.9–24.7

^a^ Data for non-ICU LOS was calculated as the difference between the outcomes “duration of hospitalization” and “duration of ICU stay” from the BAT product registry analysis.

CI, confidence interval.

Cost inputs were obtained from the literature and inflated to 2018 estimated cost values using the US Medical Care consumer price index [23] to estimate total per patient mean daily costs associated with non-ICU LOS, ICU LOS, and MV duration. Non-ICU LOS was calculated as the average cost per inpatient day for all hospital types in Becker’s Hospital Review [24]. Intensive care unit (ICU) LOS cost was calculated as the average daily cost for survivors who did not receive MV and had ICU LOS of 2 days and 5 days based on data retrieved from ICUTracker, a US commercial ICU database [25]. Mechanical ventilation (MV) cost was calculated as the incremental average daily cost for MV over no MV for patients in the ICU for 2 days and 5 days, to avoid double counting costs [25]. These values are reported in Table 2.

**Table 2:** Cost inputs—per patient mean daily costs of clinical outcomes.

Clinical Outcome Parameter	Mean Daily Cost^a^ (2018 USD)	Source
**Non-ICU LOS**	$2,265	Becker's Hospital Review 2016 [24]
**ICU LOS (no MV)**	$3,116	Kramer 2017 [25]
**MV Duration**	$1,511	Kramer 2017 [25]

^a^ All costs were inflated to 2018 values

## Results

The analysis demonstrated per patient costs were substantially higher for the late treatment group across all measured clinical parameters. Mean total cost per patient was 2.5 times higher for patients treated with BAT product late versus early. At a US population level, this results in large cost differences with administration of BAT product treatment within 2 days. Early BAT product treatment of all cases could lead to lower total costs by more than $3.9 million (versus late treatment) on average for all cases per year. Results of the analyses are reported in Table 3.

**Table 3:** Total mean costs per patient and per population for each treatment timing group.

Clinical Outcome Parameter	Early BAT Product Treatment	Late BAT Product Treatment
	Mean Cost (95% CI)	Mean Cost (95% CI)
**Total Hospital LOS**	$32,037 ($20,712 - $43,362)	$68,079 ($49,419 - $86,738)
** Non-ICU LOS**	$9,635 ($6,269 - $13,001)	$19,586 ($17,508–21,663)
** ICU LOS**	$22,402 ($14,443 - $30,361)	$48,493 ($31,911 - $65,075)
**MV Duration**	$5,570 ($3,172 - $7,967)	$26,144 ($14,916 - $37,372)
**Total Mean Cost per Patient**	$37,607 ($23,884 - $51,329)	$94,223 ($64,336 - $124,110)
**Total Population Mean Cost per Year**	$2,594,858 ($1,648,013 - $3,541,704)	$6,501,369 ($4,439,152 - $8,563,587)

CI, confidence interval.

## Discussion

Significant differences in improved clinical outcomes in patients with botulism who are treated early versus late with BAT product were previously reported [18]. The clinical benefit associated with rapid antitoxin treatment is consistent with the mechanism of action for BAT product which binds circulating toxin to prevent uptake into nerve endings thereby reducing the severity and progression of symptoms from botulinum toxin. This analysis suggests that, in addition to the demonstrated clinical benefits, substantial economic savings can be achieved in the US with early BAT product treatment versus late treatment. By reducing inpatient LOS and MV duration, early BAT product treatment is estimated to cost $2.6 million versus $6.5 million per year for late treatment, based on the average number of non-infant cases in the US. This equates to 60% higher costs for late versus early treatment. In an extreme event, such as mass exposure to botulism toxin, this calculation demonstrates a hypothetical cost if there was limited treatment supply and/or delayed access.

In the retrospective study by Payne *et al*., BabyBIG reduced mean LOS from 5.7 to 2.2 weeks for all patients who received antitoxin treatment within seven days of hospital admission compared to a placebo group. The greatest reduction in LOS was seen in patients treated earlier in their hospital course (i.e., hospital day 0 to 3); as time to treatment increased, so did the LOS [20]. Likewise, our analysis showed LOS reductions (with accompanying costs) were time-dependent.

While this analysis focused on the reductions observed in inpatient LOS and duration of MV, data from the BAT product registry also showed a difference in the need for MV itself between those patients who received BAT product early versus late. Within the early BAT product treatment group, 46.7% of patients needed MV compared to 67.7% within the late BAT product treatment group. An *ad hoc* analysis of this registry data showed the difference to be statistically significant (chi-square test, p-value = 0.029) [19]. In the real-world setting, this would appreciably reduce the strain on a local healthcare system’s resources and help protect patients without access to facilities with this type of equipment. While greater use of MV incurs costs itself, patients who require MV also face increased risks of complications such as pneumonia, pulmonary edema, and/or acute respiratory distress syndrome [26]. If prolonged airway access is necessary, a tracheostomy may be required [27]. These complications could have further consequences, including morbidity and mortality, impacts on quality of life, and significant additional healthcare costs [27].

The World Health Organization (WHO) considers botulism one of the most important foodborne illnesses globally [28] and views outbreaks as public health emergencies [3]. There is also potential for *C*. *botulinum* neurotoxins to be used in biological attacks which would cause unexpected spikes in incidence. In fact, the CDC categorizes botulism as a high-priority Category A agent similar to anthrax and smallpox [29]. High-priority agents are “organisms that pose a risk to national security because they can be easily disseminated or transmitted from person to person; result in high mortality rates and have the potential for major public health impact; might cause public panic and social disruption; and require special action for public health preparedness” [29]. In the case of mass exposure (natural or intentional), there would be considerable pressure on medical resources to manage the outbreak response. Multiple concurrent cases could significantly strain a local healthcare system’s resources; this is particularly true in under-resourced areas, although availability of ventilators and trained personnel to manage patients may be limited even in developed areas. The failure to treat early could result in severe health and economic consequences, as results of this analysis and prior studies have shown [18, 19].

There were limitations associated with this study. Only a few clinical outcomes were included; however, they represented the best data available to conduct the analyses. Additional information such as resource utilization or patient-reported outcomes would enhance the calculations and provide meaningful results on the impact to patients. Clinical outcomes were obtained from a retrospective registry study, which was the best available source of data regarding hospital resource utilization. The observational design included the risk of bias associated with factors that may have influenced whether patients were treated early versus late. However, because botulism is a rare illness, conducting a prospective, randomized study would be an extremely difficult undertaking. Furthermore, the clinical benefits of early administration of antitoxin for botulism compared with delayed administration is supported by retrospective, observational studies; indeed, the WHO and the CDC recommend administration of antitoxin as soon as possible following a clinical diagnosis of this life-threatening disease [3, 18, 19].

Costs were not associated with the same patient sample as clinical outcomes, but rather identified from multiple sources via literature review. In addition, the identified hospitalization costs were not specifically associated with botulism care. Botulism-specific cost values associated with comprehensive care including testing, intensive care such as respiratory support, and administration of antitoxin treatment, were unavailable in the published literature; thus, appropriate *per diem* proxies were used.

To explore how results were impacted across the range of clinical outcome values observed in the BAT product registry, calculated confidence intervals were used in a sensitivity analysis. Conclusions were found to be robust to variation in parameter values. Despite limitations, the results demonstrate that major economic benefits can be achieved with early treatment relative to late treatment.

This research supports the recommendation that botulism antitoxin be administered to patients as early as possible [3]. In order for this to occur, the diagnosis must be made swiftly in cases with a high index of suspicion, i.e., for patients presenting with symmetrical descending weakness or paralysis accompanied by difficulties with vision, breathing, or swallowing [7]. Confirmatory diagnostic testing for botulism takes several days to complete and has low sensitivity, so pre-emptive treatment is recommended if botulism is clinically suspected [4, 30].

Similar concerns regarding timing and availability of treatment are seen with other serious exposures such as with rabies virus, where the consequences of not receiving immediate treatment are dire [31]. The WHO reports 80% of human deaths from rabies occur in rural areas due to limited or non-existent knowledge of and access to post-exposure prophylaxis and treatment [32]. As with botulism, prompt treatment prior to confirmed diagnosis is recommended due to the severe and life-threatening manifestations of the disease [31].

It is important for public health planning to include considerations around the early availability of antitoxin for patients with botulism. The findings of this analysis support the recommendation for public health authorities to ensure antitoxin treatment is readily accessible in case of botulism outbreaks, including sporadic outbreaks and potential biological attacks, as even minor delays in providing treatment may have a significant impact on outcomes and costs on a patient or a population level.

## Disclaimer

Emergent BioSolutions, BAT^®^, and any and all Emergent BioSolutions Inc. brand, product, service and feature names, logos, and slogans are trademarks or registered trademarks of Emergent BioSolutions Inc. or its subsidiaries in the United States or other countries. All other brand, product, service and feature names or trademarks are the property of their respective owners.

## Ethics statement

This post-marketing registry study was considered a non-research public health activity that did not require informed consent by the FDA Center for Biologics Evaluation and Research (CBER) because BAT product had received FDA marketing approval and its use in clinical practice was not considered investigational in nature. No identifiable patient information was used or available for the purpose of this cost analysis study. All patient data in the registry was de-identified and a patient number was assigned by Emergent BioSolutions’ Pharmacovigilance Department before release to the Clinical Research Department, which analyzed the registry data. This number served as the patient identifier in the registry and in the registry database. The Pharmacovigilance Department follows the HIPAA Privacy Act that ensures the confidentiality and security of protected health information when it is transferred, received, handled, or shared.
